# Diversity of Environmental *Escherichia coli* in Subtropical Freshwater Systems of South Africa

**DOI:** 10.1007/s00284-025-04402-y

**Published:** 2025-07-28

**Authors:** Tarren Seale, Volker S. Brözel, Sarah C. Potgieter, Oliver Rupp, Jochen Blom, Emma T. Steenkamp, Stephanus N. Venter

**Affiliations:** 1 Department of Biochemistry, Genetics and Microbiology, University of Pretoria, Pretoria, 0002 South Africa; 2 Department of Biology and Microbiology, South Dakota State University, Brookings, SD 57007 USA; 3 Bioinformatics and Systems Biology, Justus-Liebig-University Giessen, 35390 Giessen, Germany; 4 Department of Civil and Environmental Engineering, University of Michigan, Ann Arbor, MI 48105 USA

## Abstract

*Escherichia coli* is widely used as an indicator of faecal contamination, as it is assumed that faeces from warm-blooded animals is the primary source of *E. coli* in the environment. However, various studies have shown that *E. coli* can survive and multiply in environmental niches, including soil, sand and sediment. *E. coli* can be assigned to eight main phylogroups but environmental *E. coli* are associated primarily with phylogroups A and B1. In this study, 410 *E. coli* were isolated from different niches within two peri-urban catchments in the Gauteng province of South Africa. To represent the *E. coli* circulating within the human population, *E. coli* was also isolated from sewage before treatment and released into these reservoirs. To capture the diversity of *E. coli* among these isolates, the β-D-glucuronidase (*uidA*) and mismatch repair (*mutS*) genes were sequenced. While isolates linked to phylogroup B1 dominated as was expected, the recovery of many isolates linked to phylogroup B2, the second most dominant group, was unanticipated. To further investigate this observation, the genomes of representative phylogroup B2 isolates were sequenced and subjected to phylogenetic and functional analyses. The results showed that phylogroup B2 isolates formed a distinct phylogenetic cluster, apparently independent of niche or geographic origin. Our findings also showed that while isolates seem to be associated with environmental samples, they could have the ability to colonize the gut and cause disease as they harboured several virulence factors associated with extraintestinal *E. coli*.

**Supplementary Information:**

The online version contains supplementary material available at 10.1007/s00284-025-04402-y.

## Introduction

*Escherichia coli* are frequently isolated from environments outside the gastro-intestinal tract of warm-blooded animals, where they occur as commensals [1, 2]. Populations of this bacterium can persist and become naturalized in environments ranging from soil, sand, water and wastewater to plant-associated niches—referred to as “environmental” *E. coli* [3, 4]. Because the composition of these persistent extraintestinal populations and their potential health impacts is not well understood, the interpretation of data related to the use of *E. coli* as an indicator of faecal contamination is often debated [1].

In an effort to understand the population structure and related functions of *E. coli,* a phylogenetic approach based on Multi-Locus Sequence Type (MLST) and genome data is widely used to link strains to specific lineages [5]. According to this framework, *E. coli* can be subdivided into eight phylogroups (A, B1, B2, C, D, E, F and G), which broadly correspond to different sets of ecological traits and lifestyles [5, 6]. Phylogroups A and B1 consist of commensal *E. coli,* while phylogroup B1 is also associated with pathogenic *E. coli* [6]. Phylogroup C consists of avian pathogenic *E. coli* (APEC) and Enterotoxigenic *Escherichia coli* (ETEC) [7]. Phylogroup D consists of uropathogenic *E. coli* (UPEC), enteroaggregative *E. coli* (EAEC) and extraintestinal pathogenic *E. coli* (ExPEC) [7]. Phylogroup E consists of enterohemorrhagic *E. coli* (EHEC), phylogroup F consists of ExPEC and environmental strains and phylogroup G consists of avian pathogenic *E. coli* (APEC) [7]. Phylogroup B2 includes ExPEC, UPEC and APEC. This group exhibits genetic variability and is believed to have diverged early during the evolutionary history of *E. coli* [6].

Among the known phylogroups, environments not associated with humans or animals are usually dominated by phylogroups B1 and A [3, 8–13]. For example, these two phylogroups constituted approximately 70% of the strains from drinking water in a Brazilian study, where the remaining strains mostly belonged to phylogroup D (26.5%), with phylogroup B2 being the least prevalent [12]. Similar patterns of dominance of phylogroups A and B1 have also been reported in studies focussing on surface water from the Yeongsan River basin in South Korea and from selected locations in Minnesota and Wisconsin, USA [10, 11]. Some authors have even suggested that phylogroup B1 is especially adapted to extraintestinal environments, thus explaining its dominance in plant-associated niches [3], freshwater beach sites in Michigan, USA [13] and soil collected near the Buffalo River in Minnesota, USA [9].

Human and wastewater isolates are associated mainly with phylogroups B2 and D, members of these phylogroups are known pathogens that are specifically adapted to the gastro-intestinal tract of warm-blooded animals [14]. For example, phylogroup B2 has a range of metabolic capabilities and adaptations that increase its ability to colonize the intestinal mucosa and persist in the human gut [15]. The notion that these bacteria are less fit in extraintestinal environments is consistent with their low frequency of occurrence in samples from freshwater and plant-associated environments [3, 12]. When phylogroups B2 or D occur at relatively high frequencies in freshwater and plant-associated environments, their incidence is linked to possible faecal contamination [8, 16]. In a study of surface water from the main watershed in and around Athens, Georgia, USA, phylogroup B2 was among the dominant lineages of *E. coli* detected, which the authors suggested was due to exposure of the water to faecal contamination of human and animal origin [8].

Environmental *E. coli* were initially reported from warmer tropical and subtropical regions but have since also been isolated from temperate regions [17]. However, only a limited number of these previous studies considered phylogroup associations among environmental *E. coli* populations. These include studies of samples collected from North America, South America, Europe, Asia and Australia in regions with temperate climates [1, 10], continental to humid continental climates [9, 11, 13, 18] and humid subtropical climates [8, 12, 18]. In Africa, the diversity and phylogroup distribution of environmental *E. coli* has received limited attention. The main focus of previous South African studies was on the microbial quality of vegetables and irrigation water, particularly with respect to faecal contamination using *E. coli* as indicator [16, 19]. The genetic composition and health risks associated with environmental *E. coli* populations from various aquatic environments in peri-urban regions with a subtropical climate have not been reported before. This is important information for the management of health risks associated with these environments. The aims of our study were to (i) determine the diversity of *E. coli* within two peri-urban freshwater catchments, (ii) examine how the phylogroups of isolates compare with each other and published data, (iii) investigate the genetic structure of populations using two protein-coding gene sequences and (iv) evaluate the potential virulence of isolates based on genome sequence information.

## Materials And Methods

### Sample Collection, *E. coli* Isolation and Phylogroup Assignment

The samples were obtained from the Roodeplaat dam and Rietvlei dam catchments in the Crocodile River Basin, Gauteng, South Africa. The Roodeplaat dam catchment was sampled from the inflowing Hartbees spruit (water, plant and sediment), outflow from the Zeekoegat and Baviaanspoort sewage plants that ultimately flow into the dam where we collected surface water, sediment, hyacinth, plant and algae samples. The Rietvlei dam catchment was sampled from the Olifantsfontein sewage treatment plant, which releases its effluent into the dam where we collected surface water, sediment, plants and plant debris samples. To represent *E. coli* circulating within local human populations, sewage samples were collected before sewage treatment and at final release into these reservoirs. All the samples were transported to the laboratory on ice and processed within 24 h.

Membrane Lactose Glucuronide Agar (MLGA) (Oxoid) was used to isolate *E. coli*. This medium contains indicators for β-glucuronidase (blue) and β-galactosidase (yellow) activity, enabling the differentiation of *E. coli* which exhibits activity for both, resulting in the production of green colonies. The liquid samples were diluted one thousand-fold, after which 100 μL of each dilution was plated onto MLGA and incubated at 37 °C overnight. For samples that did not yield green colonies, 1 mL was passed through 0.45 µm nitrocellulose filters, which were then placed on MLGA and incubated overnight at 37 °C. For the plant samples, a section of the plant was cut and placed into 50 mL of sterile Ringer’s solution (calcium chloride hexahydrate, 0.12 g/L; potassium chloride, 0.105 g/L; sodium bicarbonate, 0.05 g/L; and sodium chloride, 2.25 g/L) in a tube. This was sonicated for 1 min; thereafter, 10 mL was filtered through sterile 0.45 μm nitrocellulose filters (Whatman®, Merck) and placed on MLGA. For all presumptive *E. coli* isolates, β-glucuronidase and β-galactosidase activities were confirmed based on their ability to hydrolyse, 4-methylumbelliferyl-β-D-glucuronide (MUG) and ortho-nitrophenyl-β-D-galactopyranoside (ONPG) [20]. These isolates were inoculated into 5 mL of Colilert ®-18 medium (Dehteq), followed by incubation at 37 °C for 18 h and then checking for fluorescence under ultraviolet illumination.

For phylogroup assignment, genomic DNA was extracted from overnight MLGA cultures using the Quick-DNA™ MiniPrep Kit (Zymo Research). The isolates were classified into the major phylogroups A, B1, B2 or D using a PCR-based method developed by [21], with minor modifications. Briefly, this approach targets the *chuA* and *yjaA* genes, as well as the TspE4.C2 DNA fragment and modifications included the use of three separate PCR reactions rather than multiplex PCR, as well as 35 rather than 30 PCR cycles. Additionally, isolates negative for all three PCRs were denoted as “unassigned”, as suggested by [18].

### Analyses of the *uidA *and *mutS* Sequences

The DNA sequences of the two protein-coding genes were determined. These genes included the β-D-glucuronidase-encoding (*uidA)* gene*,* which is specific to *E. coli*, and the *mutS* gene, which encodes the “mismatch recognition” protein of the DNA methyl-directed mismatch repair system in *E. coli* [22]. Portions of the genes were amplified using protocols provided by the Michigan State University MLST database (http://shigatox.net/ecmlst/protocols/index.html) [22], each 25 μL reaction mixture consisted of 25 mM MgCl_2_ (Separation Scientific), 2.5 mM of each dNTP (Thermo Fisher Scientific), 5 U/μL Super-Therm *Taq* DNA polymerase with reaction buffer (Separation Scientific), 10 mM of each primer (Table S1) and 50–100 ng template DNA. The PCR conditions comprised of initial denaturation at 94 °C for 10 min, followed by 30 cycles of denaturation at 92 °C for 1 min, annealing at 60 °C for 1 min and extension at 72 °C for 1 min, followed by a final extension step at 72 °C for 5 min. A negative control containing no DNA was included for each PCR reaction to ensure no cross-contamination occurred. Amplicons were visualized using 1% (w/v) agarose gel electrophoresis and GelRed (Biotium) according to the manufacturer’s specifications. Products in the expected size range (i.e. 658 bp for *uidA* and 596 bp for *mutS*) were purified enzymatically by incubating the PCR products at 37 °C for 15 min using 20 U/μL Exonuclease I (Thermo Fisher Scientific) and 2 U/μL FastAP Alkaline Phosphatase (Thermo Fisher Scientific) followed by inactivation of the enzymes at 80 °C for 15 min. The purified products were then sequenced using the BigDye™ Terminator v3.1 Cycle Sequencing Kit (Applied BioSystems) and the ABI3500xl Genetic Analyzer (Applied Biosystems). Consensus sequences were assembled for each amplicon using BioEdit version 7.0.9.0 [23]. Each sequence dataset was aligned using ClustalW multiple alignment [23]. The aligned *uidA* and *mutS* nucleotide datasets were then concatenated and subjected to phylogenetic analyses. The dataset also included sequences downloaded from GenBank for representatives of phylogroups A, B1, B2, D and E, as well as *Shigella boydii*, *S. dysenteriae*, *S. flexneri*, *S. sonnei* and *E. coli* Clade 1, which could be considered distinct *E. coli* phylogroups [22, 24]. Other *Escherichia* species (*E. marmotae* and *E. ruysiae*) were included for outgroup purposes [22]. A maximum likelihood phylogenetic tree was inferred from the concatenated dataset using PhyML 3.0 [25], and the best-fit evolutionary model parameters were determined via jModelTest software v. 0. 1. 1 and the Akaike Information Criterion [26].

The concatenated *uidA* and *mutS* nucleotide dataset containing only the *E. coli* strains obtained from the Roodeplaat and Rietvlei dams was subjected to two sets of population genetic analyses. First, population differentiation was explored using Weir and Cockerham’s [27] *θ*. For this purpose, the concatenated dataset was subjected to MULTILOCUS v. 1.3b [28], where the null hypothesis of no population differentiation was tested by calculating *θ* across different isolate collections using 1,000,000 randomizations. These collections were defined in such a way as to evaluate population differentiation between samples from the two dams as well as between and among phylogroups (with and without accounting for sample type or sample location). The second set of population genetic analyses utilized a Bayesian clustering approach in which isolates were modelled as having a proportion of their genomes derived from one or more source populations. Analyses were performed with the programme STRUCTURE 2.3.4 following the recommendations of Wang [29]. Accordingly, independent runs involving 500,000 Markov chain Monte Carlo iterations were used following a burn-in of 10,000 and the software’s default parameters, with the only exception being the use of an *α* value of 1/K (assuming the number of clusters). The optimum K was determined by STRUCTURE HARVESTER, which compared the data (obtained for 10 independent runs) at each analysis of K, from K = 1 to K = 10. The data were processed for the 10 runs at the optimal K with CLUMPP, after which CLUMPAK was used to graphically display the results [30].

### Genome-Based Analyses

Due to their higher than expected occurrence (see below), nine presumptive phylogroup B2 isolates were selected for genome-based analyses. This selection was based on the *uidA* and *mutS* phylogenetic tree where we sought to span the genetic diversity among phylogroup B2 isolates. Accordingly, high-quality DNA was extracted from the nine isolates using the cetyltrimethylammonium bromide (CTAB) method described previously [31]. DNA quality and quantity were evaluated using a NanoDrop 2000 spectrophotometer (Thermo Fisher Scientific). These DNAs were then subjected to whole-genome sequencing using the Ion Torrent™ PGM Platform with the PI chip at the Central Analytical Facilities of the University of Stellenbosch. The genomes were assembled using IonGAP and annotated using the GenDB 2.4 Standalone pipeline [32, 33]. To confirm the phylogroup assignments for the nine isolates, they were subjected to ClermonTyping (http://clermontyping.iame-research.center/).

The annotated genome sequences generated in this study were added to a dataset containing information for other *E. coli* isolates obtained from GenBank. These were included to span the diversity of phylogroup B2 but also included genomes for representatives of each of phylogroups A, B1, D, E and F, as well as one representative from phylogroup C. The dataset additionally included genome sequences for isolates from other *Escherichia* species, as well as *E. coli* Clade 1. The entire dataset was analysed using the EDGAR 2.0 platform [34] to identify the genes common to all the genomes (i.e. core genes) and those encoded by only some of the genomes (i.e. accessory genes). The non-core genes were determined by removing the core genes from the accessory gene dataset.

For the identified core and non-core genes, dendrograms were constructed to examine the relationships among the isolates. In the case of the core gene data, individual amino acid sequences were aligned using the MUSCLE plugin in the QIAGEN CLC Main Workbench 21.0 [35]. The aligned core gene sequences were then concatenated and partitioned using FASconCAT-G 1.02 after which ProtTest 3.4 was used to determine the best-fit substitution model for each gene [36]. Maximum likelihood phylogenetic analysis was then conducted in RAxML using the identified model parameters. Branch support was estimated using the same parameters and bootstrap analysis of 1000 pseudoreplicates [37]. In the case of the non-core genes, a UPGMA (Unweighted Pair Group Method with Arithmetic Mean) tree was constructed from the gene presence/absence data using PAST3 (Paleontological Statistics Software Package for Education and Data Analysis) with the Jaccard similarity index [38].

Three rounds of pangenome analyses were conducted, with the first involving a comparison of each phylogroup B2 isolate sequenced here with known B2 strains to identify genes unique to each isolate. The amino acid sequences encoded by these unique genes were then subjected to GO-FEAT (Gene Ontology-Functional Enrichment Annotation Tool) analysis to determine their Gene Ontology (GO) terms for functional annotation [39]. The second pangenome analysis was conducted in the same way, but each of our isolates was compared with all of the strains included in the dataset. Where relevant, Fisher’s exact tests implemented in OmicsBox were used to investigate gene enrichment [40]. The third round of analyses involved the identification of virulence genes in the sequences for *E. coli* (i.e. those for *E. ruysiae*, *E. marmotae* or *E. albertii* were excluded). For this purpose, the annotation provided by EDGAR was used to determine which of the 58 virulence genes associated with ExPEC [41], eight genes linked to human association [42], and 44 UPEC and EHEC virulence genes also commonly associated with wastewater-specific strains [4] were encoded by each isolate. The presence of typical ExPEC pathovar virulence genes was also confirmed by submitting the newly sequenced genomes to the VirulenceFinder webtool on the Center for Genomic Epidemiology (CGE) website (https://cge.food.dtu.dk/services/VirulenceFinder/) or, if necessary, identifying the genes using local blast searches. The presence of the *uspC-IS30-flhDC* markers associated with wastewater-specific strains [4] was also investigated.

The database of the CGE was used to determine sequence types (STs) for the 64 isolates included in the genome dataset [43, 44]. The CGE database includes data for two MLST schemes based on eight and seven genes [43, 44]. We determined STs according to both schemes by subjecting the sequences to analyses with the MLST 2.0 tool provided on the CGE website (https://cge.food.dtu.dk/services/MLST/).

The assembled genome sequences were deposited under accession numbers JBFQXY000000000 (R2F1.2), JBFQYD000000000 (14m2), JBFQYF000000000 (4m4), JBFQYA000000000 (Q02H13), JBFQYB000000000 (Q02H4), JBFQYC000000000 (15m2), JBFQYG000000000 (1m6), JBFQYE000000000 (13m5), JBFQXZ000000000 (Q09A12) into the GenBank database (BioProject PRJNA1139092).

## Results

### Phylogroups B1 and B2 Dominate the Sampled *E. coli* Populations

A total of 410 *E. coli* isolates were obtained from the samples collected during this study (Table S2). A few strains belonging to related species have been shown to have β-glucuronidase activity [20]. Any such isolates were excluded from the study by confirming the identity of *E. coli* isolates based on their *uidA* and *mutS* sequences. There were 87 water isolates obtained from the Rietvlei (25) and Roodeplaat (62) dams. The sediment samples yielded 49 *E. coli* isolates, 31 and 18 from the respective dams. A total of 36 isolates were obtained from aquatic plants sampled from the dams, with 11 from Rietvlei and 25 from Roodeplaat, which included 13 isolates from water hyacinths, whereas plant debris (decaying plant material) sampled from the Rietvlei dam provided 24 *E. coli* isolates. A total of 172 isolates were obtained from raw sewage, with 87, 48 and 37 isolates originating from samples collected at the Olifantsfontein, Zeekoegat and Baviaanspoort treatment plants, respectively.

Application of the original Clermont et al. [21] method, allowed for separation of the 410 isolates into four main phylogroups: A (17%), B1 (38%), B2 (27%) and D (18%) (Table 1). According to this method, when amplifications of *chuA* and TspE4.C2 are negative, the isolate is assigned to phylogroup A. However, according the Gordon et al. [18] approach, isolates negative for these two markers and positive for *yjaA* are designated as phylogroup A, while isolates negative for all three markers were scored as “unassigned” (U). Therefore, according to the Gordon et al. [18] approach, the 410 isolates were separated as follows: A (8%), B1 (38%), B2 (27%), D (18%) and U (9%). In terms of overall catchment, which includes all sample types, phylogroup B2 comprised 25% of the Roodeplaat dam isolates and 29% of the Rietvlei dam isolates (Table 1). Phylogroup B1 comprised 42% and 33% of the isolates from the Roodeplaat and Rietvlei dams, respectively.

**Table 1:** Comparison of the phylogroup distribution of 410 isolates obtained from the Roodelplaat and Rietvlei dam catchments with those reported in previous studies

Sample source	Number of isolates per phylogroup					Percentage of isolates per phylogroup				
	A*	B1	B2	D	U	A*	B1	B2	D	U
Roodeplaat dam (overall)	17 (44)	97	57	34	27	7 (19)	42	24	15	12
Environmental (total)	7 (23)	79	27	18	16	5 (16)	54	18	12	11
*Algae*	0 (3)	17	3	5	3	0 (11)	60	11	18	11
*Aquatic Plant*	0 (0)	9	3	0	0	0 (0)	75	25	0	0
*Water*	3 (14)	20	5	9	11	6 (29)	42	10	19	23
*Water hyacinth*	0 (0)	0	13	0	0	0 (0)	0	100	0	0
*Sediment Hartbeesspruit*	1 (1)	14	2	1	0	6 (6)	77	11	6	0
*Algae Hartbeesspruit*	1 (3)	8	0	3	2	7 (21)	57	0	22	14
*Water Hartbeesspruit*	2 (2)	11	1	0	0	14 (14)	79	7	0	0
Sewage (total)	10 (21)	18	30	16	11	12 (25)	21	35	19	13
*Baviaanspoort*	5 (9)	9	12	7	4	14 (25)	24	32	19	11
*Zeekoegat*	5 (12)	9	18	9	7	10 (25)	19	37	19	15
Rietvlei dam (overall)	17 (27)	59	52	40	10	10 (16)	33	29	22	6
Environmental (total)	11 (18)	31	17	25	7	12 (20)	34	19	27	8
*Aquatic Plant*	2 (5)	1	4	1	3	18 (45)	9	37	9	27
*Water*	2 (6)	14	5	0	4	8 (24)	56	20	0	16
*Sediment*	5 (5)	9	7	10	0	16 (16)	29	23	32	0
*Plant debris*	2 (2)	7	1	14	0	8 (8)	29	5	58	0
Sewage (Olifantsfontein)	6 (9)	28	35	15	3	7 (11)	32	40	17	4
Both dams (overall)	34 (71)	156	109	74	37	8 (17)	38	27	18	9
Environmental (total)	18 (41)	110	44	43	23	7 (17)	46	19	18	10
Sewage (total)	16 (30)	46	65	31	14	9 (17)	27	38	18	10
Johnson et al. [11]										
Animal	54	159	51	19	–	17	51	16	6	–
Surface water	17	160	55	31	–	6	57	20	11	–
Hamelin et al. [2]										
Non-pathotypes	57	129	21	1	–	27	62	10	1	–
Pathotypes	7	10	49	34	–	7	10	49	34	–
Berthe et al. [1]										
Bovine	7	118	0	13	–	5	86	0	9	–
Human	30	4	15	1	–	60	8	30	2	–
Water	55	69	8	18	–	34	42	5	11	–
Walk et al. [13]										
Freshwater beach	43	106	12	29	–	23	56	6	15	–
Dusek et al. [9]										
Soil	0	249	110	104	–	0	43	19	18	–
Méric et al. [3]										
Plant	26	44	8	22	–	25	42	8	21	–
Cho et al. [8]										
Surface water	33	153	157	0	–	7	31	32	0	–
Orsi et al. [48]										
Recreational water	72	32	8	21	–	54	24	6	16	–
Orsi et al. [12]										
Drinking water	15	19	2	13	–	31	39	4	26	–
Jang et al. [10]										
River water	1724	1192	118	376	–	50	34	5	11	–

*Values in brackets indicate totals and percentages if the unassigned phylogroup was assigned as phylogroup A according to Clermont et al. [21]. This is because strains were typed as belonging to phylogroup A when they were negative for *chuA* and TspE4.C2 in the triplex method, but they are typed as “unassigned” when negative for all the markers in the triplex method [18]

Among the isolates from the environmental samples (i.e. non-sewage samples), 19% were assigned to phylogroup B2, whereas 46% represented phylogroup B1. More specifically, in the Roodeplaat dam catchment, 54% of the environmental isolates formed part of phylogroup B1, and 18% formed part of phylogroup B2, whereas only 5% formed part of phylogroup A (or 16%, if all the “unassigned” isolates were included with this phylogroup). The same pattern was observed in the Rietvlei catchment samples, where most of the environmental isolates were typed as belonging to phylogroups B1 (34%) and B2 (19%), and phylogroup A contained only 12% of the environmental isolates (20% if the latter included the “unassigned” isolates). Phylogroup B2 (38%) was the dominant group linked to the sewage isolates as well as the aquatic plants with a high percentage of 55%. In contrast, the most dominant phylogroup associated with the plant debris was group D at 58%.

### Phylogroup B2 is Genetically Distinct Based on *uidA *and* mutS* Sequences

To explore relatedness and evaluate the genetic diversity among the *E. coli* isolates obtained from the two catchments, the nucleotide sequences of *uidA* and *mutS* were analysed. Phylogenetic analyses of the concatenated dataset separated the 410 isolates into various clusters, with no clear patterns reflecting the source of the isolates (Supplementary Fig. S1). However, individual clusters mostly contained isolates from a particular phylogroup, which was particularly evident for phylogroups B2 and D. With respect to the environmental samples, isolates were spread across the tree, although most of them grouped with isolates from phylogroups B1 and B2.

Analysis of the concatenated *uidA* and *mutS* data with MULTILOCUS yielded highly significant but low *θ* values when comparing isolates from the Roodeplaat dam catchment with those from the Rietvlei dam catchment (*θ* = 0.012*, p* = 0.0014) or when comparing phylogroup B2 isolates from the two catchments (*θ* = 0.031*, p* = 0.0104). Such values are indicative of little population differentiation, suggesting that the two isolate collections essentially form part of the same overall population Wright (1978 as cited in [45]). This contrasts with the much stronger genetic differentiation detected (*θ* = 0.3068*, p* < 0.0001) when the analysis was performed using isolate collections defined based on their phylogroup assignments.

**Fig. 1:**
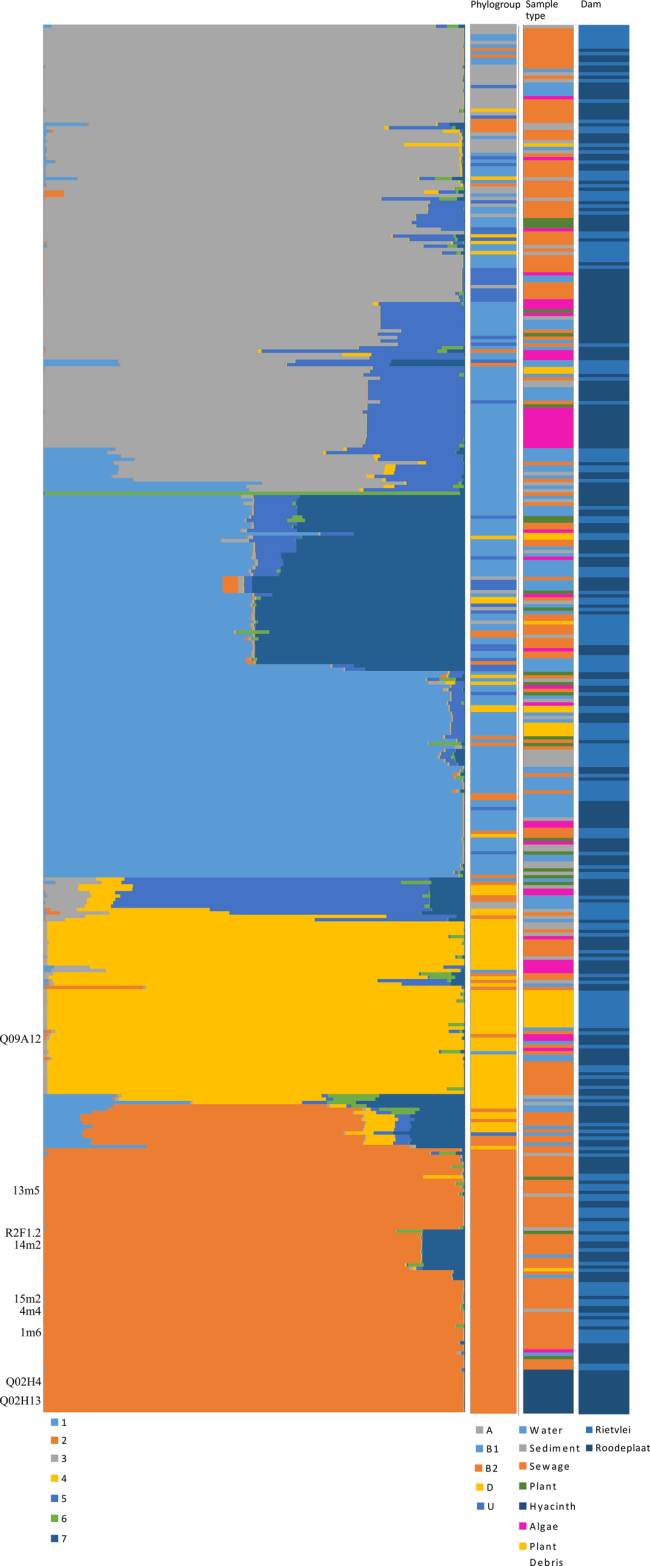
Population structure analysis of *E. coli* isolates. Concatenated *mutS* and *uidA* sequences were analysed under the assumption of the presence of two populations, but analysis using Structure Harvester revealed that K = 7 best explained the data. Each population was assigned a colour, and each isolate was assigned to one or more inferred source or ancestral populations making up 100% of the population. The graphical display of population structure was ordered according to the phylogenetic tree (Fig. S1). The nine sequenced isolates from this study are indicated on the left of the figure and include three aquatic plant (R2F1.2, Q02H4 and Q02H13), one algal (Q09A12) and five isolates (14m2, 4m4, 1m6, 13m5 and 15m2). Phylogroups are indicated according to the Gordon et al. [18] approach

The observed genetic patterns were supported by the results of the STRUCTURE analysis of the concatenated *uidA* and *mutS* sequences (Fig. 1). This Bayesian approach for investigating population structure suggested that the genomes of the included isolates were derived from seven source populations (K = 7). However, the inferred ancestry proportions of the isolates did not correlate with their geographic origins or the sample types from which they were recovered. Instead, the inferred ancestry of the isolates reflected their phylogroup assignments, especially in the case of phylogroups B2 and D. For the latter, the largest proportion of the genomes of isolates from the respective phylogroups were each derived from single source populations (i.e. orange for phylogroup B2 and yellow for phylogroup D in Fig. 1). For phylogroups A, B1 and U, such a clear separation was lacking, as, irrespective of phylogroup assignment, ancestry membership for the isolates included one or combinations of four main source populations (i.e. grey, light blue, green and two darker blue populations), two of which were more dominant. Nevertheless, many exceptions to these patterns were also evident, with isolates from a particular phylogroup sharing ancestry membership with isolates from another phylogroup.

### Genome Data Cluster Environmental and Sewage Isolates of Phylogroup B2 Together

The nine bacteria for which genome sequences were generated were selected to represent phylogenetic diversity among our B2 isolates (Fig. S1) and included three aquatic plant (R2F1.2, Q02H4 and Q02H13), one algal (Q09A12) and five isolates (14m2, 4m4, 1m6, 13m5 and 15m2) from sewage samples. Their genome assemblies consisted of 84 to 221 contigs totaling approximately 5 Mb in size (Table S4), ranging from 4.81 Mb for isolate Q09A12 to 5.37 Mb for isolate R2F1.2. Confirmation of their initial phylogroup assignments using the genome-based ClermonTyping scheme further confirmed that all isolates except isolate Q09A12 formed part of phylogroup B2. This isolate was assigned to phylogroup G, which is closely related to B2 [5]. All nine of these new sequences were included in a larger dataset containing the genome sequences for 55 reference strains (i.e. phylogroups A, B1, B2, C-F and *E. coli* Clade 1, as well as *E. marmotae*, *E. ruysiae*, *S. boydii*, *S. dysenteriae*, *S. flexneri* and *S. sonnei*). Some of these genomes were complete, consisting of a single contig, whereas others contained as many as 1936 contigs (see Table 2 and Table S4 for details regarding their origins and genomes). Analysis with EDGAR allowed the identification of 1537 genes common to all 64 genomes included in the dataset (i.e. core genes), whereas 22,550 genes were missing in one or more of the genomes (i.e. non-core genes).

**Table 2:** Pathotype, source, GenBank accession number and MLST1 and MLST2* sequence types of the isolates and reference strains used

Strain	Phylogroup	Pathotype^#^	Source	Accession number	MLST 1	MLST 2
*E. coli* UTI89	B2	ExPEC	Human	NC_007946	ST-95	ST-1
*E. coli* UM146	B2	AIEC	Human	NC_017632	ST-643	ST-1
R2F1.2	B2		Plant	JBFQXY000000000	ST-95	ST-1
*E. coli* PMV1	B2	ExPEC	Human	NC_022370	ST-6134	ST-1
*E. coli* IHE3034	B2	ExPEC	Human	NC_017628	ST-95	ST-1
*E. coli* S88	B2	ExPEC	Human	NC_011742	ST-95	ST-1
14m2	B2		Sewage	JBFQYD000000000	ST-95	ST-1
*E. coli* APECO1	B2	ExPEC	Avian	NC_008563	ST-95	ST-418
*E. coli* ECOR64	B2	ExPEC	Human	LYDC00000000	ST-14	ST-6
4m4	B2		Sewage	JBFQYF000000000	ST-1193	ST-53
*E. coli* F11	B2	ExPEC	Human	AAJU02000001	ST-127	ST-33
*E. coli* 536	B2	ExPEC	Human	NC_008253	ST-127	Unknown
Q02H13	B2		Hyacinth	JBFQYA000000000	Unknown	Unknown
Q02H4	B2		Hyacinth	JBFQYB000000000	Unknown	Unknown
15m2	B2		Sewage	JBFQYC000000000	ST-372	ST-455
*E. coli* ED1a	B2	Commensal	Human	NC_011745	ST-452	ST-149
*E. coli* TA014	B2	Commensal	Potoroo	ADKC00000000	ST-104	ST-798
*E. coli* clone Di14	B2	ExPEC	Human	NC_017652	ST-73	ST-4
*E. coli* CFT073	B2	ExPEC	Human	NC_004431	ST-73	ST-4
*E. coli* ABU 83972	B2	ExPEC	Human	NC_017631	ST-73	ST-4
*E. coli* ECOR57	B2	Commensal	Gorilla	LYCX00000000	ST-73	ST-4
*E. coli* ECOR60	B2	Commensal	Human	LYDA00000000	ST-12	ST-36
*E. coli* J96	B2	ExPEC	Human	ALIN02000001	ST-12	ST-36
*E. coli* H223	B2	EAEC	Human	ADIV00000000	ST-141	ST-10
*E. coli* LF82	B2	AIEC	Human	NC_011993	ST-135	ST-64
*E. coli* O83:H1 NRG 857C	B2	AIEC	Human	NC_017634	ST-135	ST-64
1m6	B2		Sewage	JBFQYG000000000	ST-1170	Unknown
*E. coli* ECOR66	B2	ExPEC	Human	LYDE00000000	ST-83	ST-207
*E. coli* NA114	B2	ExPEC	Human	NC_017644	ST-131	ST-43
*E. coli* JJ1886	B2	EIEC	Human	NC_022648	ST-131	ST-43
*E. coli* SE15	B2	Commensal	Human	NC_013654	ST-131	ST-506
13m5	B2		Sewage	JBFQYE000000000	ST-131	ST-506
*E. coli* O127:H6 E2348/69	B2	EPEC	Human	NC_011601	ST-15	ST-491
Q09A12	G		Algae	JBFQXZ000000000	ST-657	ST-385
*E. coli* O7:K1 CE10	F	ExPEC	Human	NC_017646	ST-62	ST-254
*E. coli* IAI39	F	ExPEC	Human	NC_011750	ST-62	ST-254
*E. coli* UMN026	D	ExPEC	Human	NC_011751	ST-414	Unknown
*E. coli* 042	D	EAEC	Human	NC_017626	ST-597	ST-3
*S. dysenteriae* 1617				NC_022912	ST-146	Unknown
*S. dysenteriae* Sd197				NC_007606	ST-146	Unknown
*E. coli* O55:H7 CB9615	E	EPEC	Human	NC_013941	ST-335	ST-553
*E. coli* O157:H7 EDL933	E	EHEC	Human	NC_002655	ST-11	Unknown
*E. coli* H10407	A	ETEC	Human	NC_017633	ST-48	ST-132
*E. coli* P12b	A	EPEC	Human	NC_017663	ST-10	Unknown
*S. flexneri* 5 8401				NC_008258	ST-634	Unknown
*S. flexneri* 2a 301				NC_004337	ST-245	Unknown
*E. coli* 55,989	B1	EAEC	Human	NC_011748	ST-678	ST-290
*E. coli* O111:H^−^ 11,128	B1	EHEC	Human	NC_013364	ST-16	ST-480
*E. coli* APECO78	C	APEC	Avian	NC_020163	ST-23	ST-708
*S. sonnei* Ss046				NC_007384	ST-152	ST-563
*S. sonnei* 53G				NC_016822	ST-152	ST-563
*S. boydii* CDC 3083–94/BS512				NC_010658	ST-1129	Unknown
*S. boydii* Sb227				NC_007613	ST-1130	Unknown
*Escherichia* TW15838	Clade I			NZ_AEJX00000000	ST-3692	Unknown
*Escherichia* TW10509	Clade I			GL872204	ST-747	ST-675
*E. ruysiae* TW09276				NZ_AEJV00000000		
*E. ruysiae* TW09231				NZ_AEJW0000000		
*E. ruysiae* H605				NZ_ADJX00000000		
*E. ruysiae* TW14182				NZ_AEJZ00000000		
*E. marmotae* E1118				NZ_ADKG00000000		
*E. albertii* NIAH				AP014855		
*E. albertii* CB9786				AP014856		
*E. albertii* EC06				NZ_AP014857		
*E. fergusonii* ATCC 35469				NC_011740		

*MLST 1 according to Wirth et al. [44] and MLST 2 according to Jaureguy et al. [43]

^#^*ExPEC* extraintestinal *E. coli*, *AIEC* adherent-invasive *E. coli, EIEC* enteroinvasive *E. coli*, *EPEC* enteropathogenic *E. coli*, *EAEC* enteroaggregative *E. coli*, *EHEC* enterohemorrhagic *E. coli, ETEC* enterotoxigenic *E. coli* and *APEC* Avian pathogenic* E. coli*

**Fig. 2:**
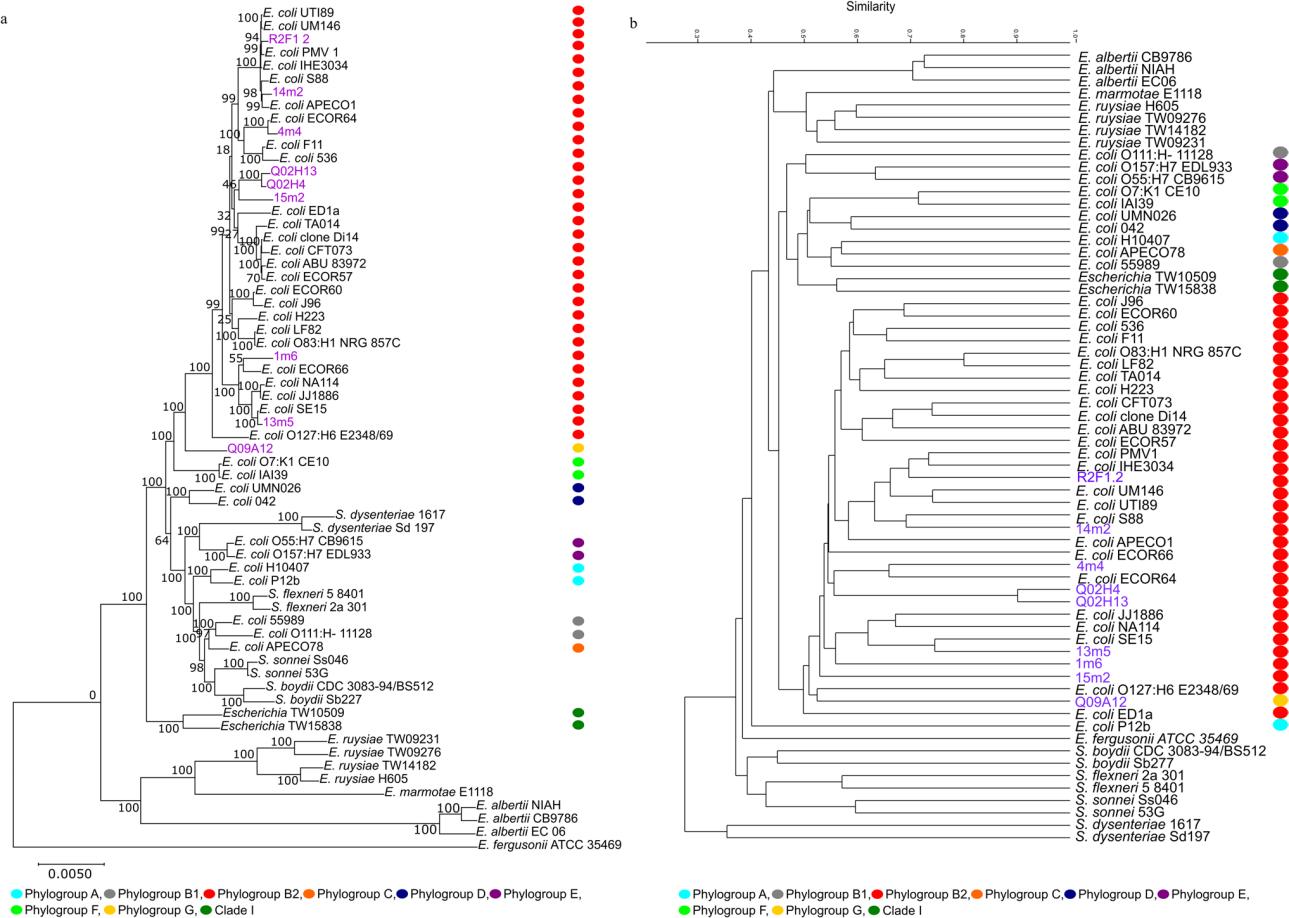
Core and non-core phylogenetic analysis. **a** The amino acid core genome tree using 1537 core genes determined by EDGAR 2.0, drawn using RAxML. The tree is rooted using *E. fergusonii*, branch support based on 1000 bootstrap is indicated at the nodes and the scale bar depicts substitutions per site. **b** The amino acid non-core genome dendrogram using 22,550 genes determined by EDGAR 2.0, drawn using PAST3. For both of these analyses, the 55 reference phylogroups used, as well as the nine isolates from this study, are denoted using the phylogroup key and isolates from this study are indicated in purple

RAxML-based Maximum likelihood analysis of the concatenated data for the corresponding amino acid sequences of the core genes revealed that phylogroup B2 formed a cohesive cluster despite its high diversity (Fig. 2a). All the phylogroup B2 isolates were clustered together with 100% bootstrap support. The sister-group relationship between phylogroup B2 and phylogroup G isolate Q09A12 received 100% bootstrap support. Similarly, the PAST3-based UPGMA dendrogram inferred from the presence/absence data for the 22,550 non-core genes clustered all phylogroup B2 isolates together at approximately 50% similarity, with isolate Q09A12 nested within it (Fig. 2b). However, no clear patterns were evident in either the core or non-core gene trees in terms of the sampling origins of the isolates. In other words, our environmental- and sewage-related isolates were scattered across the respective clusters containing phylogroup B2 isolates. The only exceptions were isolates Q02H4 and Q02H13, which originated from water hyacinths in the Roodeplaat dam and always grouped together as part of a unique lineage.

The presence/absence analysis of the virulence genes (non-core), typically associated with ExPEC (Mahfouz et al. 2018) also supported the phenotypic cohesiveness among phylogroup B2 isolates irrespective of their source (Fig. S2). Despite the large diversity observed among phylogroup B2 isolates, most contained 20 or more of these genes, suggesting that they likely represent ExPEC (Mahfouz et al. 2018). Additionally, seven of our nine isolates had 20 or more virulence genes, of which three were aquatic plant-associated (R2F1.2, Q02H4 and Q02H13) and four originated from sewage strains (14m2, 4m4, 1m6 and 13m5). The two strains with fewer than 20 virulence genes originated from sewage (15m2), whereas the other represents an environmental strain isolated from algae (Q09A12), which was confirmed to belong to phylogroup G.

In addition, virulence genes among wastewater-specific isolates were explored to determine whether these genes were present in the *E. coli* strains used in this study. All phylogroup B2 isolates had genes associated with iron acquisition except for the reference strain ED1a *(fepA*) (Fig. S2). Selected genes typically associated with UPEC strains were present in most of the B2 isolates. None of these isolates had the virulence genes associated with EHEC isolates and the *uspC-IS30-flhDC* maker associated with wastewater-specific strains was absent among all the B2 isolates [4].

Exploration of the functional properties encoded by the phylogroup B2 genomes also supported the close association of the environmental and sewage isolates examined in the present study. This was particularly evident for genes encoding products involved in adaptation to human-associated niches, such as the UmuDC SOS DNA polymerase*,* EmrYK and EvgAS efflux systems, the BetTB chlorine transporter and the NhaR transcriptional activator of the proton-sodium antiport system [42]. Regardless of the samples from which they originated, the genomes of all phylogroup B2 isolates encoded *umuDC, emrYK* and *evgAS*. All phylogroup B2 isolates also contained the *betB* and *betT* genes, with our sewage isolate 15m2 and reference strain ED1a being the only exceptions. Similarly, the *nhaR* gene was present in all the isolates except for the reference strain F11 (Fig. S2).

### Sequence Types and Inferred Gene Functions of Environmental Isolates

GO-FEAT analysis was used to explore the functions encoded by the genes that are unique to each of our algae and plant-associated (R2F1.2, Q02H4, Q02H13 and Q09A12) or sewage genomes (14m2, 4m4, 1m6, 13m5 and 15m2). Here, “unique genes” refer to those present in the genome of a particular isolate relative to those encoded by the genomes of the remaining 55 strains included in our genome dataset (see Table 2). The number of functional categories in the strains ranged from 19 for strain Q02H13 to four for strain 14m2. Analyses based on Fisher’s exact test of the unique genes in all the isolates revealed that isolate 1m6 had genes that were significantly over-represented. These genes had “molecular function” GO terms involved in the catalysis of biochemical reactions (GO:0003824, *p* = 2.5 × 10^–6^) and in binding/interaction with one or more specific sites on another molecule (GO:0005488, *p* = 1.7 × 10^–5^), as well as the “cellular component” GO term involved in cellular anatomical structure (GO:0110165, *p* = 3.2 × 10^–5^).

Overall, the complement of genes unique to each of the nine genomes had only the “carbohydrate metabolism” functional category in common. The GO terms associated with this category also made up a large proportion of the functional capability of the unique genes (Fig. S3). The “stress response” category also appeared to be an important functional capability, although GO terms associated with this category were not included among the genes unique to isolate R1F1.2 obtained from the water hyacinths. However, the plant-associated isolates generally lacked GO terms associated with “virulence, disease and defence”, which were present in three of the sewage isolates (Fig. S3).

Seven of the nine isolates examined represented known sequence types (STs) in one or both of the MLST schemes (1 and 2) proposed by Wirth et al. [44] and Jaureguy et al. [43], respectively. For those reference strains for which this information is lacking, we also determined their STs (see Table 2). Our environmental isolate R2F1.2 and sewage isolate 14m2 were both assigned to ST-95 in Scheme 1 and to ST-1 in Scheme 2. Sewage isolate 13m5 was assigned to ST-131 in Scheme 1 and ST-506 in Scheme 2. Another four of the nine isolates were assigned to STs, with 4m4 representing ST-1193 (scheme 1) and ST-53 (scheme 2), 15m2 representing ST-372 (scheme 1) and ST-455 (scheme 2), and Q09A12 representing ST-657 (scheme 1) and ST-385 (scheme 2). Sewage isolate 1m6 represented ST-1170 in scheme 1 and UNKNOWN in scheme 2. Additionally, the water hyacinth-associated isolates Q02H13 and Q02H4 could not be assigned to known STs using either scheme, indicating that they belong to hitherto undescribed sequence types.

## Discussion

Here, we describe for the first time the prevalence and diversity of environmental *E. coli* from subtropical freshwater catchments in Southern Africa. Application of the widely used Clermont et al. [21] method revealed that populations from the Rietvlei and Roodeplaat dam catchments were dominated by phylogroups B1, B2 and D, where they accounted for 46%, 19% and 18%, respectively, of the isolates obtained from the surface water, sediments, algae, plants and plant debris. Phylogroup B1 typically dominates non-host environments, as strains from this phylogroup are capable of persisting and thriving in water environments [3, 12, 13]. However, the dominance of phylogroup B2 and D associated with aquatic plant and plant debris samples, respectively, was unexpected, as they are usually associated with extraintestinal infections [3, 12, 13]. Phylogroups B2 and D are reportedly rare in non-host environments, where they lose culturability after introduction [46].

Higher than expected numbers of phylogroup B2 isolates were recovered from the aquatic plant-associated samples collected in the two freshwater catchments targeted. This phylogroup constituted 19% of all the environmental isolates. The phylogroup B2 levels of the water- and sediment-associated samples corresponded with levels observed in other freshwater systems [1, 8, 11]. However, 24% of the isolates from the aquatic plant-associated samples represented phylogroup B2 (see Table S3), which is substantially higher than that previously reported for plant-associated *E. coli* [3]. The reasons underlying this pattern are unclear; the phylogroup B2 strains predominant in plant-associated samples may be able to persist on the surfaces of aquatic plants. These plant-associated phylogroup B2 strains which persisted on the surface of the aquatic plants, especially the water hyacinths, could have been introduced from the human reservoir by passage through sewage treatment into the dams or could represent plant-specific ecotypes [47]. Generally, the detection of *E. coli* in aquatic environments would indicate a recent faecal contamination event, but the finding that these isolates can persist on the surface of aquatic plants may affect the use of *E. coli* as an indicator of faecal contamination [12].

A small proportion of the *E. coli* detected in the present study formed part of phylogroup A, although its distribution varied among samples. This was similar to reports for environmental samples in North America [8, 11] but much lower than other reports for freshwater environments, where phylogroup A is more abundant and even dominant [1, 3, 10, 12, 13, 48]. This was true, irrespective of whether the three-marker triplex PCR method or the four-marker quadruplex PCR method was used for typing strains. A strain is typed as belonging to phylogroup A when it is negative for *chuA* and TspE4.C2 via the triplex method; however, when the three markers were analysed separately, the strain was typed as unknown when *chuA, yjaA* and TspE4.C2 were not present [18, 21]. In the present study, 7% and 10% of the environmental isolates were typed as belonging to phylogroup A and to an unassigned phylogroup, respectively, via the quadruplex PCR system. However, even when the triplex PCR system was used, the abundance of phylogroup A among our environmental isolates was lower than that reported in many previous studies [1, 10, 12, 48].

The phylogroup distribution patterns observed among the host-associated samples (sewage) linked to the catchments examined differed markedly from those observed among the environmental samples. Within the collection of sewage samples, the majority of isolates constituted phylogroups B1 (27%) and B2 (38%), with phylogroup D accounting for 18%. The prevalence of phylogroups B2 and D was expected, as they are closely associated with animals/humans and often cause extraintestinal infections [3, 12, 13]. Phylogroups B2 and D are associated with wastewater as well as the intestinal microbiota of humans; however, phylogroup B2 is dominant and has increased persistence in these sample types [1, 15]. Nevertheless, a substantial proportion of the *E. coli* population in the sewage samples was also represented by phylogroup B1, which is normally associated with environmental samples and might reflect the high abundance of these bacteria in the overall system.

Phylogenetic and population genetic analyses of the concatenated nucleotide sequences of the *mutS* and *uidA* genes revealed that the *E. coli* populations in the Rietvlei dam and Roodeplaat dam catchments were genetically diverse yet highly interconnected. When comparing the *mutS* and *uidA* phylogeny with the genome trees, the genome trees were able to distinguish the phylogroups more accurately. Based on the *θ* statistic Weir and Cockerham [27], which is equivalent to Wright’s *F*_*ST*_ (1978 as cited in [45]), a *θ* value of 0.25 and above is suggestive of strong genetic variation, as was the case for our comparisons among phylogroups. Populations displaying little to no differentiation has *θ* values of 0–0.05, which was the case for our comparisons involving all isolates from the two catchments (*θ* = 0.012) and phylogroup B2 isolates from the two catchments (*θ* = 0.031). These values suggest that *E. coli* isolates within these catchments are highly mobile within these aquatic environments and probably also the human communities in the area. Bayesian analysis suggested that the variation observed in our collection of *E. coli* isolates was most likely derived from as many as seven different genetic backgrounds or source populations [49]. Based on their ancestry membership, phylogroup B2 and D isolates were more differentiated from one another and from those representing phylogroups A, B1 and U. This is consistent with the higher recombination rates previously observed between members of phylogroups A and B1 than between these groups and phylogroups B2 and D or between the latter two groups [50]. This interconnectedness of phylogroups A and B1 is also consistent with the notion that they represent recently diverged sister lineages [6, 50]. In contrast, the strong differentiation between phylogroups B2 and D is consistent with a much earlier origin during *E. coli* evolution [6]. Therefore, despite varying levels of gene flow being maintained among phylogroups, their differentiation (particularly among phylogroups B2, D and A + B1) likely forms the basis of niche adaptation [50].

Because of the unexpected prevalence of phylogroup B2 among environmental samples, particularly those associated with algae and plants, we subjected several strains to various genomic analyses. Phylogenetic analyses of datasets containing conserved genes included in the core genome and more variable genes included in the non-core genome of *E. coli* and its close relatives grouped our B2 isolates with known members of this phylogroup. Also, the maximum likelihood phylogeny inferred from the 1537-core-gene dataset robustly grouped all *E. coli* into a single clade with each of the phylogroups clearly separated from one another and from *E. coli* Clade I, with the four *Shigella* species (i.e. *S. flexneri, S. dysenteriae, S. boydii* and *S. sonnei*, all regarded as being conspecific to *E. coli*) nested among them [24, 51]. Although similar groupings have been reported in previous studies, some did not recover all of the phylogroups as monophyletic [50, 52]. Additionally, in the study by Meier-Kolthoff and colleagues [52], *Shigella* did not group according to species but was scattered among other *E. coli* strains. Nevertheless, both the core and non-core gene trees emphasized the phylogenetic cohesion among members of phylogroup B2, with our environmental and sewage isolates clustering with known members of the phylogroup, independent of niche or geographic origin. This suggests that these *E. coli* isolates are highly mobile within these aquatic environments. Phylogroup B2 is usually human associated and based on this observation our isolates linked to environmental sources could be part of the *E.coli* population circulating in the community inhabiting this larger catchment area.

Analysis of the putative functions encoded by the respective *E. coli* genomes suggested that our environmental and sewage-associated phylogroup B2 isolates likely share adaptive and clinical properties with known members of the phylogroup. All but one of our phylogroup B2 isolates encoded 20 or more of the 58 known virulence genes, suggesting that they might represent ExPEC [41]. Indeed, phylogroup B2 typically includes ExPEC isolates and is well adapted for host colonization to cause extraintestinal infections [6]. The presence of several virulence factors associated with UPEC [4] confirmed this finding as the UPEC strains are considered to be a specific disease associated group belonging to ExPEC [53]. Furthermore, genome-based comparisons against international databases using the Center for Genomic Epidemiology (CGE) revealed that most of our phylogroup B2 isolates represent known STs in both commonly used MLST schemes for *E. coli* [43, 44]. For example, our isolates were diagnosed as ST-95, ST-1193, ST-372, ST-1170 and ST-131 according to the Wirth et al. [44] scheme. ST-95 is usually associated with neonatal meningitis and contains the UPEC PAI marker *malX* [54], as well as with cell attachment and invasion in human and rat brain cell lines [54]. Additionally, certain ST-131 and ST-1193 strains have been reported to be multidrug resistant [14], whereas ST-372 is associated with dogs and causes infrequent human infections [55]. However, we analysed only eight phylogroup B2 isolates, and it would be interesting to determine whether any one of the 46 remaining isolates available from the current study represent STs implicated in diseases such as those reported by Vignaroli et al. [56] of *E. coli* from extraintestinal disease. Additionally, it would be interesting to determine if and how the novel STs detected are implicated in disease. Overall, there was no distinction among our B2 isolates in terms of their geographic origin or source, again emphasizing that they form part of the larger population of phylogroup B2 isolates.

The cohesiveness among phylogroup B2 isolates, including our eight isolates (isolate Q09A12 was not included in this analysis as it was assigned to phylogroup G), was also supported by numerous shared sets of genes and processes encoded by these bacteria. For example, their accessory genomes contain genes previously associated with colonization of the gut, as well as genes needed for stress responses [42]. Based on the unique genes identified in the sequenced isolates, a subset of virulence, disease and defense genes was found exclusively in the sewage-derived isolates, but absent in the environmental isolates. Defense genes play a crucial role in helping bacteria evade the host immune system. Their absence from the non-core genomes of environmental strains may suggest a reduced capacity for gut colonization. In contrast, most Phylogroup B2 isolates, regardless of whether they originated from sewage or aquatic environments, contained a variety of virulence genes, particularly those associated with ExPEC. While these genes may indicate pathogenic potential, Zhi and colleagues noted that such genes might also provide advantages for extraintestinal survival [4]. The persistence of *E. coli* Phylogroup B2 strains in the environment, particularly those associated with aquatic plants, raises concerns about the reliability of *E. coli* as an indicator of potential health risks. However, the virulence of these environmental isolates warrants further validation.

## Conclusions

A higher than expected proportion of phylogroup B2 was detected in the *E. coli* population from the peri-urban subtropical catchments examined, although phylogroup B1 was predominant. Phylogroup B2 showed a distinct grouping based on both core and non-core genes. Genome analyses suggested that our isolates could be associated with human sources. Two of our sequenced isolates were related to multidrug resistant strains and two to strains attaching to human and rat brain cell lines. Seven of our isolates were also denoted as ExPEC and UPEC. Association of many of our B2 strains with water plants and algae indicate persistence in the catchment, with the risk of circulating back into the human population and manifesting disease. Furthermore, the occurrence of isolates that lack unique virulence, disease or defence genes, and isolates of unknown sequence type such as those associated water hyacinth, raise the possibility of adaptation to aquatic environments and population maintenance on plant surfaces. These findings highlight the complexities of using *E. coli* as an indicator organism, particularly in environmental systems influenced by treated wastewater inputs. Its presence in freshwater catchments does not necessarily signify contamination, underscoring the need for more nuanced interpretations and further investigations when *E. coli* is detected in such contexts.

## Supplementary Information

Below is the link to the electronic supplementary material.

Supplementary file1 (TIFF 383288 kb)

Supplementary file2 (TIFF 474825 kb)

Supplementary file3 (TIFF 344675 kb)

Supplementary file4 (DOCX 38 kb)

## Data Availability

The datasets presented in this study can be found in online repositories as follows: https://www.ebi.ac.uk/ena/, PRJEB35465; https://www.ncbi.nlm.nih.gov/, PRJNA1139092.
