# Supplementary material for: Diversity of Environmental Escherichia coli in Subtropical Freshwater Systems of South Africa
Source: Curr Microbiol. 2025 Jul 28;82(9):414. doi: 10.1007/s00284-025-04402-y (PMC12304041; doi:10.1007/s00284-025-04402-y)
Supplement: Supplementary file 4 — Supplementary file4 (DOCX 38 kb) [file 284_2025_4402_MOESM4_ESM.docx]

**Supplementary Material**

**Table S1.** Primers used in amplification and Sanger sequencing of mutS and uidA

| Gene | Forward primer sequence | Reverse primer sequence |
| --- | --- | --- |
| *uidA* | CAT TACGGCAAAAGTGTGGGTCAAT | TCAGCGTAAGGGTAATGCGAGGTA |
| *mutS* | GGCCTATACCCTGAACTACA | GCATAAAGGCAATGGTGTC |

**Table S2.** Isolate names and sample types in the two catchments

| Isolate name | Sample type | Catchment | GPS coordinates |
| --- | --- | --- | --- |
| TS1B, TS2A, TS2B, TS3A, TS3B, TS4B, TS5A, TS5B, TS6A, TS6B, TS7A, TS7B, TS8B, TS9A, TS10A, TS11A, TS12B, TS13A, TS14B, TS15A, TS15B, TS16A, TS17A, TS17B, TS18A | Dam water | Rietvlei Dam | -25°53'29.39"S, 28°17'22.80E |
| KW3, KW4, KW5, KW6A, KW6B, KW7, KW8, KW9, KW10, KW11, KW12A, KW12B, KW13, KW15, Q011, Q013, Q021, Q023, Q024, Q025, Q026, Q027, Q072, Q073, Q076, Q077, Q078, Q0710, Q083, Q084, Q085, Q086, Q087, Q088, Q0810, Q0811, Q0812, Q0813, Q091, Q093, Q094, Q095, Q096, Q097, Q098, Q099, Q0910, Q0911, Q0912, Q0913, Q0914, Q0915, Q105, Q106, Q107, Q109, Q1010A, Q1010B, Q1012, Q1013, Q1014, Q1015 | Dam water | Roodeplaat Dam | 25°37′15″S, 28°22′17″E |
| S21.1G, S21.2G, S21.3G, S21.4G, S21.5G, S21.6G, S21.7G, S21.8G, S21.10G, S21.11G, S21.13G, S21.14G, S226Y, S2F1.1G, S2F1.2G, S2F1.3G, S2F1.4G, S2F1.5G, S2F1.6G, S2F1.7G, S2F1.8G, S2F1.11G, S2F1.12G, S2F1.13G, S2F1.14G, S2F1.15G, S2F2.1G, S2F2.3G, S3F2.1G, S3F2.2G, S3F2.3G | Sediment | Rietvlei Dam | -25°53'29.39"S, 28°17'22.80E |
| KS1A, KS1B, KS2, KS3, KS4, KS5, KS6, KS7, KS8, KS9, KS10, KS11A, KS11B, KS12, KS13, KS14A, KS14B, KS15 | Sediment | Roodeplaat Dam | 25°37′15″S, 28°22′17″E |
| RVD1.1, RVD2.2, RVD3.1, RVD4.1, RVD4.2, RVD5.2, RV1F1.1, RV1F1.2, RV1F1.3, RV1F2.1, RV1F2.2, RV1F2.3 | Plant | Rietvlei Dam | -25°53'29.39"S, 28°17'22.80E |
| R1F1.1, R1F1.2, R1F1.3, R1F1.5, R1F2.1, R1F2.5, R1S1.1, R1S1.2, R1S1.3, R2F1.2, R2F1.3, R2F1.4 | Plant | Roodeplaat Dam | 25°37′15″S, 28°22′17″E |
| DWWF1.2G, DWWF1.3G, DWWF1.4G, DWWF2.1G, DWWF2.2G, DWWF2.3G, DWWF2.4G, DWWF2.5G, DWWF2.6G, DWWF2.7G, DWWF2.8G, DWWF2.9G, DWWF2.10G, DWWF2.11Y/G, DWPD5.1, DWPD5.2, DWPD5.3, DWPD5.4, DWPD5.5, DWPD5.6, DWPD5.7, DWPD5.8, DWPD5.9, DWPD5.10 | Plant debris | Rietvlei Dam | -25°53'29.39"S, 28°17'22.80E |
| Q02H1, Q02H2, Q02H3, Q02H4,Q02H5, Q02H6, Q02H8, Q02H9, Q02H10, Q02H11, Q02H12, Q02H13, Q02H15 | Water Hyacinth | Roodeplaat Dam | 25°37′15″S, 28°22′17″E |
| JA1, JA2, JA3, JA4, JA5, JA6A, JA6B, JA7, JA8, JA9, JA10, JA11, JA12, JA13, JA14, JA15, KA1A, KA4, KA5, KA6, KA7, KA8A, KA8B, KA9, KA10, KA12, KA13A, KA13B, KA14, KA15, Q09A2, Q09A3, Q09A4, Q09A5, Q09A6, Q09A8, Q09A9, Q09A10, Q09A12, Q09A13, Q09A14, Q09A15 | Algae | Roodeplaat Dam | 25°37′15″S, 28°22′17″E |
| Final2.1G, Final.2G, Final 2.3G, Final 2.5G, Final 2.6G, Final 2.7G,  Final 2.8G, Final 2.8G2, Final 2.9G, Final2.10G, Final 2.15Y, Raw4.5G, Raw4.6G, Raw4.8G2, Raw4.9G2 | Sewage | Hartebeesfontein sewage works | 26°01′11.58”S, 28°17′04.80”E |
| 1m4, 2m4, 3m4, 4m4, 5m4, 6m4,7m4, 8m4, 9m4, 10m4, 11m4, 12m4, 13m4, 15m4, 16m4, 18B4, 20m4, 21m4, ZA1.3, ZA1.4, ZA1.5, ZA1.6, ZA1.7, ZA1.8, ZA1.9, ZA2.1, ZA2.2B, ZA2.3, ZA2.4, ZA2.5, ZA2.6, ZA2.7, ZA2.9, ZB1.2, ZB1.3, ZB1.5, ZB1.6, ZB1.7, ZB1.8, ZB1.9, ZB1.10, ZB2.2, ZB2.3, ZB2.4, ZB2.5, ZB2.7, ZB2.9, ZB2.10 | Sewage | Zeekoegat sewage works | 25°37′15″S, 28°22′17″E |
| 1B, 2B, 3B, 5B, BB, DB, EB, FB, IB, CB, GB, HB, 1m2, 2m2, 3m2, 4m2, 5m2, 6m2, 7m2, 8m2, 9m2, 10m2, 11m2, 12m2, 13m2, 14m2, 16m2, 17m2, 18m2, 19m2, 20m2, 21m2, 15m2, 22m2, 1m3, 2m3, 3m3, 4m3, 5m3, 6m3, 7m3, 8m3, 9m3, 10m3, 11m3, 12m3, 13m3, 14m3, 15m3, 16m3, 17m3, 18m3, 19m3, 20m3, 1m6, 2m6, 3m6, 5m6, 6m6, 7m6, 8m6, 9m6, 10m6, 11m6, 13m6, 14m6, 15m6, 16m6, 17m6, 18m6, 19m6, 20m6 | Sewage | Olifantsfontein sewage works | 25°56′24.48″S, 28°12′57.96″E |
| 1m5, 2m5, 4m5, 5m5, 6m5, 7m5, 8m5, 11m5, 12m5, 13m5, 14m5, 15m5, 16m5, 17m5, 18m5, 20m5, 21m5, 22m5, 24m5, 25m5, 26m5, B1.2, B1.3, B1.5, B1.6B, B1.7, B1.9, B1.10, B2.1, B2.2A, B2.2B, B2.3, B2.4, B2.5, B2.7, B2.8, B2.10 | Sewage | Baviaanspoort sewage works | 25°45′05.94″S, 28°22′44.16 E |

**Table S3.** The phylogroup distribution percentages of 102 plant, plant material and algae-associated isolates into the four major phylogroups A, B1, B2 and D was determined via the PCR protocol designed by Clermont et al. [21]

| **Phylogroup** | **Total** | **A*** | **B1** | **B2** | **D** | **U** |
| --- | --- | --- | --- | --- | --- | --- |
| Aquatic plant | 36 | 6(14) | 28 | 55 | 3 | 8 |
| Algae | 42 | 2(14) | 60 | 7 | 19 | 12 |
| Plant debris | 24 | 8(8) | 29 | 4 | 59 | 0 |

*Values in brackets indicate percentages if the unassigned phylogroup was assigned as phylogroup A according to Clermont et al. [21]. This is because strains were typed as belonging to phylogroup A when they were negative for *chuA* and TspE4.C2 in the triplex method, but they are typed as “unassigned” when negative for all the markers in the triplex method [18].

**Table S4.** Genome properties of the strains used in this study

| **Strain** | **Phylogroup** | **Pathotype** | **Source** | **Accession number** | **Genome size (Mb)** | **Number of contigs** | **Coverage** |
| --- | --- | --- | --- | --- | --- | --- | --- |
| *E. coli* UTI89 | B2 | ExPEC | Human | NC_007946 | 5,17 | Complete |  |
| *E. coli* UM146 | B2 | AIEC | Human | NC_017632 | 5,19 | 89 |  |
| **R2F1.2** | B2 |  | Plant | JBFQXY000000000 | 5,37 | 221 | 31,01 |
| *E. coli* PMV1 | B2 | ExPEC | Human | NC_022370 | 5,18 | Complete |  |
| *E. coli* IHE3034 | B2 | ExPEC | Human | NC_017628 | 5,11 | Complete |  |
| *E. coli* S88 | B2 | ExPEC | Human | NC_011742 | 5,17 | Complete |  |
| **14m2** | B2 |  | Sewage | JBFQYD000000000 | 5,26 | 147 | 32,11 |
| *E. coli* APECO1 | B2 | ExPEC | Avian | NC_008563 | 5,5 | 68 |  |
| *E. coli* ECOR64 | B2 | ExPEC | Human | LYDC00000000 | 5,17 | 519 |  |
| **4m4** | B2 |  | Sewage | JBFQYF000000000 | 5,05 | 93 | 30,93 |
| *E. coli* F11 | B2 | ExPEC | Human | AAJU02000001 | 5,21 | 119 |  |
| *E. coli* 536 | B2 | ExPEC | Human | NC_008253 | 4,93 | Complete |  |
| **Q02H13** | B2 |  | Hyacinth | JBFQYA000000000 | 4,88 | 144 | 123,19 |
| **Q02H4** | B2 |  | Hyacinth | JBFQYB000000000 | 4,89 | 84 | 113,89 |
| **15m2** | B2 |  | Sewage | JBFQYC000000000 | 4,88 | 173 | 31,29 |
| *E. coli* ED1a | B2 | Commensal | Human | NC_011745 | 5,21 | 2 |  |
| *E. coli* TA014 | B2 | Commensal | Potoroo | ADKC00000000 | 4,73 | 36 |  |
| *E. coli* Di14 | B2 | ExPEC | Human | NC_017652 | 5,04 | Complete |  |
| *E. coli* CFT073 | B2 | ExPEC | Human | NC_004431 | 4,72 | 94 |  |
| *E. coli* ABU 83972 | B2 | ExPEC | Human | NC_017631 | 5,13 | Complete |  |
| *E. coli* ECOR57 | B2 | Commensal | Gorilla | LYCX00000000 | 5,7 | 1440 |  |
| *E. coli* ECOR60 | B2 | Commensal | Human | LYDA00000000 | 5,04 | 325 |  |
| *E. coli* J96 | B2 | ExPEC | Human | ALIN02000001 | 5,54 | 276 |  |
| *E. coli* H223 | B2 | EAEC | Human | ADIV00000000 | 5,13 | 155 |  |
| *E. coli* LF82 | B2 | AIEC | Human | NC_011993 | 4,77 | Complete |  |
| *E. coli* O83:H1 NRG 857C | B2 | AIEC | Human | NC_017634 | 4,89 | Complete |  |
| **1m6** | B2 |  | Sewage | JBFQYG000000000 | 4,96 | 192 | 37,81 |
| *E. coli* ECOR66 | B2 | ExPEC | Human | LYDE00000000 | 5,54 | 1936 |  |
| *E. coli* NA114 | B2 | ExPEC | Human | NC_017644 | 5,25 | 105 |  |
| *E. coli* JJ1886 | B2 | EIEC | Human | NC_022648 | 5,31 | Complete |  |
| *E. coli* SE15 | B2 | Commensal | Human | NC_013654 | 4,84 | Complete |  |
| **13m5** | B2 |  | Sewage | JBFQYE000000000 | 5,13 | 106 | 31,73 |
| *E. coli* O127:H6 E2348/69 | B2 | EPEC | Human | NC_011601 | 5,07 | Complete |  |
| **Q09A12** | G |  | Algae | JBFQXZ000000000 | 4,81 | 92 | 33,77 |
| *E. coli* O7:K1 CE10 | F | ExPEC | Human | NC_017646 | 5,38 | Complete |  |
| *E. coli* IAI39 | F | ExPEC | Human | NC_011750 | 5,13 | Complete |  |
| *E. coli* UMN026 | D | ExPEC | Human | NC_011751 | 5,36 | 4 |  |
| *E. coli* 042 | D | EAEC | Human | NC_017626 | 4,79 | Complete |  |
| *S. dysenteriae* 1617 |  |  |  | NC_022912 | 4,48 | Complete |  |
| *S. dysenteriae* SD197 |  |  |  | NC_007606 | 4,56 | Complete |  |
| *E. coli* O55:H7 CB9615 | E | EPEC | Human | NC_013941 | 5,45 | Complete |  |
| *E. coli* O157:H7 EDL933 | E | EHEC | Human | NC_002655 | 5,33 | 374 |  |
| *E. coli* H10407 | A | ETEC | Human | NC_017633 | 5,33 | Complete |  |
| *E. coli* P12b | A | EPEC | Human | NC_017663 | 4,94 | Complete |  |
| *S. flexneri* 5 str 8401 |  |  |  | NC_008258 | 4,57 | Complete |  |
| *S. flexneri* 2a 301 |  |  |  | NC_004337 | 4,83 | Complete |  |
| *E. coli* 55989 | B1 | EAEC | Human | NC_011748 | 5,15 | Complete |  |
| *E. coli* O111:H^-^ 11128 | B1 | EHEC | Human | NC_013364 | 5,77 | Complete |  |
| *E. coli* APECO78 | C | APEC | Avian | NC_020163 | 4,8 | Complete |  |
| *S. sonnei* Ss046 |  |  |  | NC_007384 | 5,06 | Complete |  |
| *S. sonnei* 53G |  |  |  | NC_016822 | 5,22 | Complete |  |
| *S. boydii* CDC 3083-94/BS512 |  |  |  | NC_010658 | 4,87 | Complete |  |
| *S. boydii* Sb227 |  |  |  | NC_007613 | 4,65 | Complete |  |
| *Escherichia* TW15838 |  |  |  | NZ_AEJX00000000 | 5,27 | 517 |  |
| *Escherichia* TW10509 |  |  |  | GL872204 | 5,19 | 453 |  |
| *E. ruysiae* TW09276 |  |  |  | NZ_AEJV00000000 | 4,47 | 328 |  |
| *E. ruysiae* TW09231 |  |  |  | NZ_AEJW0000000 | 4,74 | 495 |  |
| *E. ruysiae* H605 |  |  |  | NZ_ADJX00000000 | 4,5 | 70 |  |
| *E. ruysiae* TW14182 |  |  |  | NZ_AEJZ00000000 | 4,68 | 533 |  |
| *E. marmotae* E1118 |  |  |  | NZ_ADKG00000000 | 4,63 | 75 |  |
| *E. albertii* NIAH |  |  |  | AP014855 | 4,56 | Complete |  |
| *E. albertii* CB9786 |  |  |  | AP014856 | 4,6 | Complete |  |
| *E. albertii* EC06 |  |  |  | NZ_AP014857 | 4,66 | Complete |  |
| *E. fergusonii* ATCC 35469 |  |  |  | NC_011740 | 4,64 | Complete |  |
|  |  |  |  |  |  |  |  |

**Supplementary Figure Legends:**

**Fig. S1.** Maximum likelihood tree constructed using phyML with the K2+G+I (Kimura 2-parameter with discrete gamma distribution and allowance for proportion of invariable sites) evolutionary model of the concatenated *uidA* and *mutS* genes of the 410 isolates, the phylogroups and the clades. The nine isolates selected for genome sequencing are indicated with stars.

**Fig. S2.** Distribution of virulence genes typically associated with ExPEC [41], genes encoding products involved in adaptation to human associated niches [42] and virulence genes among wastewater-specific isolates [4] across the genomes of the nine isolates sequenced in this study and the 46 genomes of the *E. coli* reference isolates. Isolates from the current study are indicated in bold, while blue squares denote the presence of virulence genes, and white squares indicate their absence. The isolates were ordered according to the core genome tree (Fig. 2a)

**Fig. S3.** Functional categories of the unique genes within the (a) sewage-associated and (b) plant-associated genomes generated using GO FEAT. Isolate Q09A12 was omitted from this analysis as this isolate was assigned to phylogroup G
